# Closing the gap: New report estimates the resources required to eliminate trachoma

**Published:** 2025-08-16

**Authors:** PJ Hooper, Michaela Kelly, Angelia Sanders

**Affiliations:** 1 Chair: International Coalition for Trachoma Control, Atlanta, USA.; 2 Vice Chair: International Coalition for Trachoma Control, Haywards Heath, United Kingdom.; 3 Immediate Past Chair: International Coalition for Trachoma Control, Atlanta, USA.

**Investment will lift the social and financial burden of trachoma.**

**Figure:**
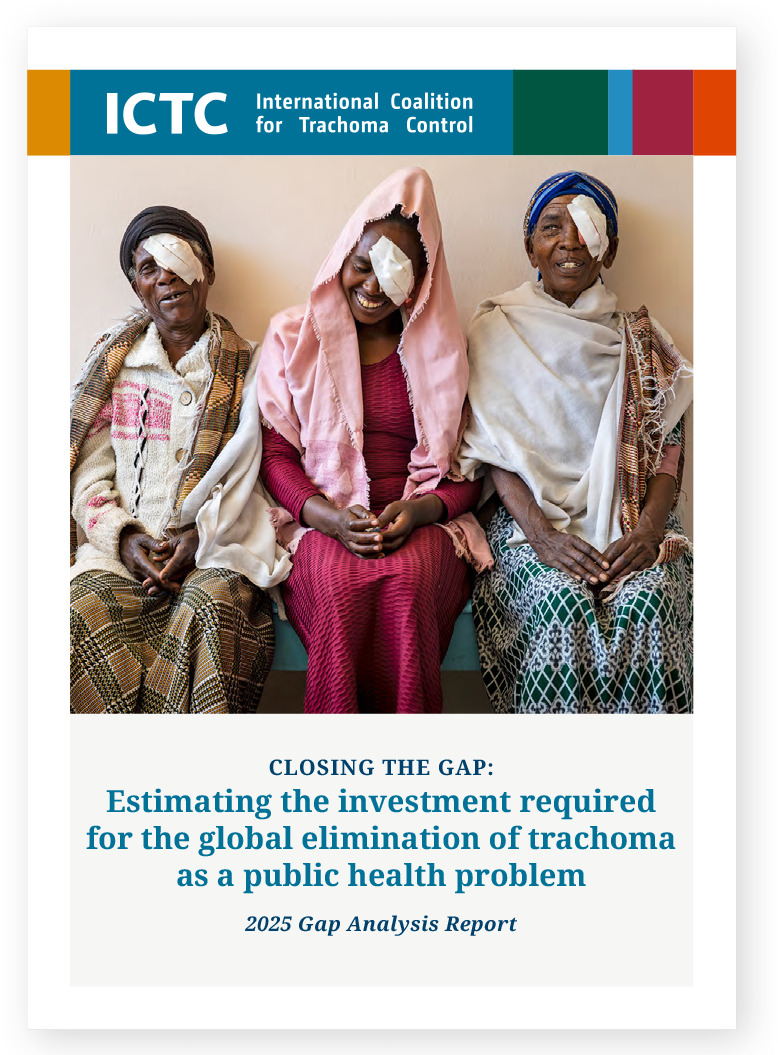
An additional US $268–334 million is needed to eliminate trachoma as a global health problem by 2030. Read more here: bit.ly/43sC2EH

Significant progress is being made toward eliminating trachoma as a public health problem. Since 2002, scale-up of the World Health Organization (WHO)-endorsed SAFE strategy (Surgery, Antibiotics, Facial cleanliness, and Environmental improvement) has contributed to a 93% reduction in the number of people at risk of trachoma and 23 countries being validated by WHO as having eliminated trachoma as a public health problem. These achievements are the result of sustained commitment from national trachoma programmes and the active engagement of affected communities, in collaboration with a global network of stakeholders –including NGOs, academic and research institutions, and donors.

To maintain momentum and ensure global elimination is achieved, the International Coalition for Trachoma Control (ICTC) published a global funding gap analysis in April 2025.^1^ The analysis outlines the additional financial commitments needed through 2030, the target year for global elimination set out in the global neglected tropical disease (NTD) road map, which was endorsed by WHO Member States at the 73rd World Health Assembly. The analysis estimates that an additional US $268–334 million is required to support trachomatous trichiasis (TT) management, antibiotic mass drug administration (MDA), surveys, and high-priority research from 2025 to 2030.

Key investment areas outlined in the report include:

**Surgery (US$105–140 million).** Although the number of people needing TT surgery – the advanced and blinding stage of the disease – is decreasing, funding is needed for an estimated 390,000 individuals requiring surgical intervention. Maintaining high-quality, accessible surgery is essential. Funding is needed to support case finding, community outreach, refresher surgical training, quality monitoring, and post-operative follow-up, all of which are crucial for ensuring patient safety and effective outcomes. Continued investment will also support the integration of TT surgery into national health systems, making services more sustainable and resilient.

**Antibiotic MDA (US $91–121 million).** Approximately 103 million people still live in areas requiring MDA for trachoma. Funding will support the delivery of antibiotics through 2030, including logistics, microplanning, and community engagement to ensure high coverage and uptake. This investment also leverages an estimated US $2.27 billion in donated antibiotics from Pfizer Inc., provided through the International Trachoma Initiative, representing one of the most significant pharmaceutical donations in global health.

**Surveys (US $63 million).** Reliable data remains central to eliminating trachoma. Investments will support both standard prevalence surveys and enhanced (‘plus’) surveys to verify progress, identify areas of residual transmission, and guide decisions about where and when to scale down interventions. These surveys are particularly important for preparing national programmes for WHO validation.

**Research (US $8.7–$9.8 million).** Operational and implementation research will address ongoing high-priority knowledge gaps. This includes exploring how to manage persistent or recrudescent active trachoma, improve MDA effectiveness, refine epidemiological indicators, assess the impact of facial cleanliness and environmental improvement interventions, and test alternative delivery models. These studies help optimise programme delivery and support the broader integration of trachoma interventions into national eye health systems.

Eliminating trachoma as a public health problem will deliver lasting impact and improve the lives of people in some of the world’s most remote and marginalised communities. It will reduce avoidable blindness and vision impairment, improve access to education and economic opportunities, and lift the social and financial burden of this disease.

Beyond immediate health benefits, investing in trachoma elimination is an investment in health system strengthening. Investing in the areas above bolsters eye health services in countries where infrastructure is limited. A range of preferred practice documents, developed by ICTC members, are available to support national programmes in strengthening human resources for eye health, guiding the transition of trachoma interventions into routine services, and embedding these practices across the health system. As a result, countries are becoming better equipped to manage a wide array of eye health challenges, helping to accelerate progress toward universal eye health coverage and the broader goal of equitable health for all.
